# Linked color imaging aids in identification of multiple rectal neuroendocrine tumors

**DOI:** 10.1055/a-2081-9749

**Published:** 2023-05-26

**Authors:** Dmitrii Dolgunov, Ker Kan Tan, Bernice Tan, Calvin Jianyi Koh, Chieh Sian Koo

**Affiliations:** 1 Division of Colorectal Surgery, Department of Surgery, University Surgical Cluster, National University Health System, Singapore; 2 Division of Gastroenterology and Hepatology, National University Hospital, Singapore; 3 Yong Loo Lin School of Medicine, National University of Singapore, Singapore

A 63-year-old man with a history of three synchronous primary cancers (colon, lung, and thyroid) underwent surveillance colonoscopy after his curative left hemicolectomy. A new 8 mm rectal lesion was identified and removed with hot snare polypectomy. Histology showed a grade 1 rectal neuroendocrine tumor (NET). Repeat colonoscopy 6 months later showed five new sub-centimeter rectal NETs, which were removed with cap-assisted endoscopic mucosal resection (EMR).

Another surveillance colonoscopy (EC-760ZP-V/L with 7000 System; Fujifilm, Tokyo, Japan) was arranged 6 months later. Assessment with standard white-light endoscopy was now challenging due to the scars from previous endoscopic resections ( Fig. 1 ). Use of linked color imaging (LCI) identified a new small (3 mm) lesion with yellowish discoloration that was not discernible on white-light endoscopy ( Fig. 2 , Video 1 ). The lesion was removed using cap-assisted EMR, and post-resection histology confirmed a well-differentiated NET.

**Fig. 1:**
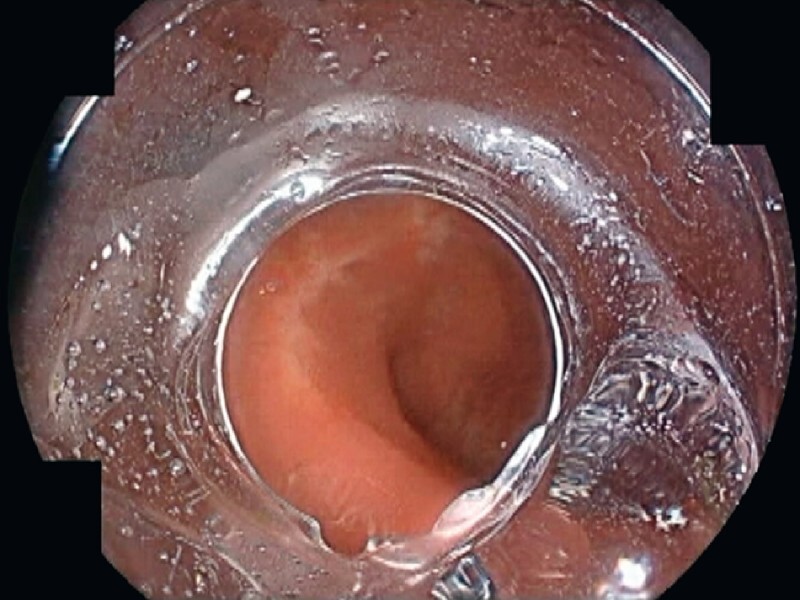
Assessment of the rectal mucosa was challenging with standard white-light endoscopy due to the scars from previous endoscopic resections.

**Fig. 2:**
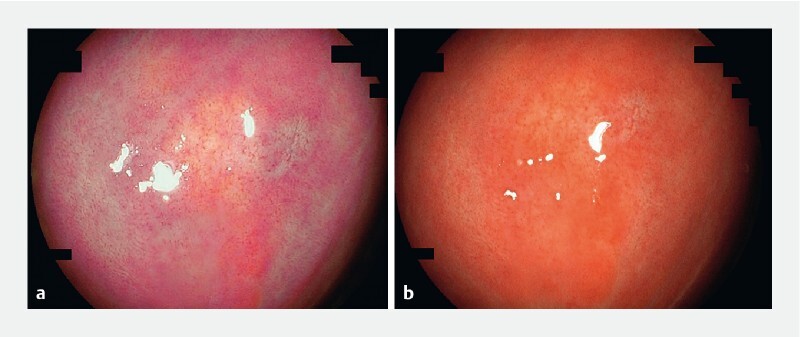
Endoscopic images of the new lesion. **a** Linked color imaging revealed a yellowish lesion. **b** The lesion was not well seen with white-light endoscopy.

**Video 1** Linked color imaging aids in identification of recurrent rectal neuroendocrine tumors.


There were also several small nodules found at previous resection sites, but it was difficult to ascertain with standard white-light endoscopy whether these were due to granulation scar tissue or NET recurrence ( Fig. 3 a ). LCI demonstrated that the nodules were purple even on magnified zoom ( Fig. 3 b ). This was suggestive of normal mucosa, and biopsies confirmed benign scar tissue.

**Fig. 3:**
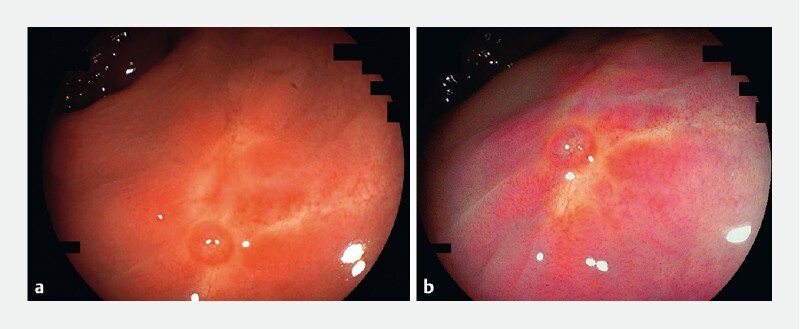
Endoscopic images of small nodules found at the previous resection sites. **a** White-light endoscopy. **b** Linked color imaging.

Rectal NETs are typically characterized as yellowish subepithelial lesions. Unfortunately, their appearance can sometimes resemble that of hyperplastic or adenomatous polyps, making endoscopic identification problematic 1 . LCI enhances the color contrast and has been shown to improve the diagnostic accuracy when detecting colorectal polyps 2 . This case describes a patient with multiple small rectal NETs that were difficult to identify with traditional white-light and image-enhanced endoscopic modes. LCI has the unique ability to better identify rectal NETs by intensifying the color difference between normal and abnormal mucosa. Endoscopists should consider the use of LCI when there is a high suspicion for NETs.

Endoscopy_UCTN_Code_CCL_1AD_2AC

## Notes

Endoscopy E-Videos

https://eref.thieme.de/e-videos
*E-Videos* is an open access online section of the journal *Endoscopy* , reporting on interesting cases and new techniques in gastroenterological endoscopy. All papers include a high-quality video and are published with a Creative Commons CC-BY license. Endoscopy E-Videos qualify for HINARI discounts and waivers and eligibility is automatically checked during the submission process. We grant 100% waivers to articles whose corresponding authors are based in Group A countries and 50% waivers to those who are based in Group B countries as classified by Research4Life (see: https://www.research4life.org/access/eligibility/ ). This section has its own submission website at https://mc.manuscriptcentral.com/e-videos
